# Effect of genotyping density on the detection of runs of homozygosity and heterozygosity in cattle

**DOI:** 10.1093/jas/skae147

**Published:** 2024-05-27

**Authors:** Laura Falchi, Alberto Cesarani, Andrea Criscione, Jorge Hidalgo, Andre Garcia, Salvatore Mastrangelo, Nicolò Pietro Paolo Macciotta

**Affiliations:** Dipartimento di Agraria, Università degli Studi di Sassari, Sassari 07100, Italy; Dipartimento di Agraria, Università degli Studi di Sassari, Sassari 07100, Italy; Department of Animal and Dairy Science, University of Georgia, Athens 30602, USA; Dipartimento di Agricoltura, Alimentazione e Ambiente, Università degli Studi di Catania, Catania 95123, Italy; Department of Animal and Dairy Science, University of Georgia, Athens 30602, USA; American Angus Association, Angus Genetics Inc., Saint Joseph, MO, USA; Dipartimento di Scienze Agrarie, Alimentari, e Forestali, Università degli Studi di Palermo, Palermo 90128, Italy; Dipartimento di Agraria, Università degli Studi di Sassari, Sassari 07100, Italy

**Keywords:** Simmental cattle, inbreeding, genomic regions, sensitivity analysis

## Abstract

Runs of homozygosity (**ROHom**) are contiguous stretches of homozygous regions of the genome. In contrast, runs of heterozygosity (**ROHet**) are heterozygosity-rich regions. The detection of these two types of genomic regions (ROHom and ROHet) is influenced by the parameters involved in their identification and the number of available single-nucleotide polymorphisms (**SNPs**). The present study aimed to test the effect of chip density in detecting ROHom and ROHet in the Italian Simmental cattle breed. A sample of 897 animals were genotyped at low density (50k SNP; 397 individuals), medium density (140k SNP; 348 individuals), or high density (800k SNP; 152 individuals). The number of ROHom and ROHet per animal (**nROHom** and **nROHet**, respectively) and their average length were calculated. ROHom or ROHet shared by more than one animal and the number of times a particular SNP was inside a run were also computed (**SNP**_**ROHom**_ and **SNP**_**ROHet**_). As the chip density increased, the nROHom increased, whereas their average length decreased. In contrast, the nROHet decreased and the average length increased as the chip density increased. The most repeated ROHom harbored no genes, whereas in the most repeated ROHet four genes (*SNRPN*, *SNURF*, *UBE3A*, and *ATP10A*) previously associated with reproductive traits were found. Across the 3 datasets, 31 SNP, located on *Bos taurus* autosome (**BTA**) 6, and 37 SNP (located on BTA21) exceeded the 99th percentile in the distribution of the SNP_ROHom_ and SNP_ROHet_, respectively. The genomic region on BTA6 mapped the *SLIT2, PACRGL*, and *KCNIP4* genes, whereas 19 and 18 genes were mapped on BTA16 and BTA21, respectively. Interestingly, most of genes found through the ROHet analysis were previously reported to be related to health, reproduction, and fitness traits. The results of the present study confirm that the detection of ROHom is more reliable when the chip density increases, whereas the ROHet trend seems to be the opposite. Genes and quantitative trait loci (**QTL**) mapped in the highlighted regions confirm that ROHet can be due to balancing selection, thus related to fitness traits, health, and reproduction, whereas ROHom are mainly involved in production traits. The results of the present study strengthened the usefulness of these parameters in analyzing the genomes of livestock and their biological meaning.

## Teaser

A total of 897 Italian Simmental animals were genotyped at different densities to investigate the impact of the BeadChip density on detection of the runs of homozygosity (ROHom) and heterozygosity (ROHet). The results suggested that ROHom detection becomes more accurate as the density increases and that ROHom and ROHet may be used to identify genomic regions mainly due to directional and balancing selection, respectively.

## Introduction

Runs of homozygosity (**ROHom**) are contiguous stretches of homozygous segments within genomes, first recognized by Broman and Weber (1999) in human populations. They reflect autozygosity (McQuillan et al., 2008) because the homozygous segments may have been inherited from common ancestors, i.e., these segments are identical by descent (Purfield et al., 2012) and can be used as a predictor of inbreeding (Ferenčaković et al., 2013a). Their length is a temporal indicator of the inbreeding occurrence (Kirin et al., 2010): recent inbreeding results in longer ROHom, whereas ancient inbreeding is associated with shorter ROHom because recombination events break the segments over each generation. The ROHom can be due to natural and artificial selection because of the fixation of homozygous favorable alleles at selected loci; linkage disequilibrium can extend the variation of allele frequency to neighboring loci, resulting in an increase of homozygosity and the proportion of the genome covered by ROHom (Macciotta et al., 2021). For this reason, ROHom shared within a population can assist in identifying breed-specific regions potentially under selection (Mastrangelo et al., 2018a). Runs of heterozygosity (**ROHet**) cannot be defined as true runs but rather as heterozygosity-rich regions (Marras et al., 2018) because they are not as dense and contiguous as ROHom are. The ROHet were first introduced by Williams et al. (2016) in a study on Chillingham white cattle, which had only 9.1% polymorphic loci compared to the 62% to 90% found in commercial cattle breeds. Interestingly, these few polymorphic loci clustered in specific chromosomal regions, named ROHet blocks, that contain genes resilient to genetic drift with possible effects on fitness (Williams et al., 2016). These heterozygous regions could be associated with survival rate, fertility, and other fitness traits (McParland et al., 2009). ROHet islands can be investigated to search for balancing selection, defined as the natural selection that maintains genetic diversity via heterozygote advantage (Fijarczyk and Babik, 2015). Since the definition of ROHet is a relatively recent concept, the literature about this metric is not as abundant as for ROHom. After the first study by Williams et al. (2016), four other studies about ROHet in cattle can be found in the literature (Ferenčaković et al., 2016; Biscarini et al., 2020; Hidalgo et al., 2021; Mulim et al., 2022). Moreover, few studies have been conducted on other livestock species, e.g., in sheep (Selli et al., 2021), goats (Li et al., 2022; Chessari et al., 2024), turkey (Marras et al., 2018), horses (Santos et al., 2021, 2023), and pigs (Chen et al., 2022; Ruan et al., 2022; Bordonaro et al., 2023), with a lack of consensus in establishing the criteria to define ROHet. Therefore, one main challenge is defining the parameters (i.e., minimum run length, number of consecutive heterozygote markers, and missing single-nucleotide polymorphisms, **SNPs**) for their correct identification. Moreover, the density of the SNP chip used is another factor affecting autozygosity and heterozygosity estimates. Together with the lack of golden standards parameters for ROHom and ROHet, the genotyping density can introduce bias in their detection.

The ROHom tend to be more abundant in inbred and strongly selected populations (Kim et al., 2018), whereas in theory, ROHet may be more common in outbred or less selected populations (Chessari et al., 2024). The latter can be identified in local and dual-purpose populations in which the rate of artificial selection is usually lower than cosmopolitan breeds.

The Italian Simmental cattle breed represents a good livestock model to investigate both ROHom and ROHet. This breed is farmed mostly in small herds in the mountainous areas of Northeastern Italy (Cesarani et al., 2020) and it is the third largest Italian cattle breed (www.vetinfo.it, Sistema Informativo Veterinario 2022). The aim of this study was to investigate the differences in the detection of ROHom and ROHet using three different BeadChip genotyping arrays and the signals of selection highlighted by the two approaches.

## Materials and Methods

Animal Care and Use Committee approval was not needed as data were obtained from preexisting databases.

### Animals and genotypic data

Three datasets were used and consisted of a total of 897 Italian Simmental cattle: 397 genotypes were obtained using the Illumina BovineSNP50k array and denoted as low density (50k SNP, **LD**), 348 genotypes were obtained using the customized Geneseek genomic profiler GGP-HDv3 and denoted as medium-density (140k SNP, **MD**), and 152 genotypes were obtained using the Illumina BovineHD and denoted as high density (800k SNP, **HD**). After quality control carried out using PLINK v. 1.9 (Purcell et al., 2007; Chang et al., 2015), SNP were retained for the analysis if: there was no statistical deviation from the Hardy–Weinberg equilibrium (*P* < 1e^−6^), minor allele frequency (**MAF**) was > 0.01, individual animal and SNP call rates were > 95%. In addition, SNP mapped on allosomes or unmapped according to the ARS-UCD1.3 were discarded. After quality control, 43,431 SNP were retained for the LD, 113,042 for the MD, and 583,637 for the HD datasets.

### Detection of runs

ROHom and ROHet were identified using the “consecutive” algorithm implemented in the “*detectRUNS*” R package (Biscarini et al., 2019). To minimize the number of false-positive ROHom, the minimum number of SNP to define an ROHom was computed using the following formula from Purfield et al. (2012):


$$\begin{array}{l} nSNP_{ROH}=\frac{\log_{e}\alpha /(n_{s}.\;n_{i})}{\log_{e}(1-het)}, \end{array}$$ (1)

where *n*_s_ is the number of SNP per individual, *n*_*i*_ is the number of individuals, α is the percentage of false positive (0.05), and het is the average heterozygosity. The same equation (1) was used to compute the minimum number of SNP that constituted an ROHet, considering homozygosity instead of heterozygosity in the denominator. In order to account for the lower number of heterozygote genotypes in the genome, the number of opposite SNP allowed in a ROHet (i.e., homozygotes inside the run) was computed as


$$\begin{array}{l} maxOpp=\;\;\;\frac{nSNP_{ROHom}}{nSNP_{ROHet}}, \end{array}$$ (2)

where *n*SNP_ROHom_ is the minimum number of SNP in an ROHom and *n*SNP_ROHet_ is the minimum number of SNP in an ROHet, both computed using equation (1).

From this, the following parameters were adopted:

(i) ROHom = 50 homozygote SNP and no heterozygote or missing allowed within the run;
(ii) ROHet = 18 heterozygote SNP, 3 opposite (i.e., homozygote), and 0 missing.

For both ROHom and ROHet, the minimum length and the maximum gap between adjacent SNP were set to 1 Mb.

The number of ROHom and ROHet per animal (**nROHom** and **nROHet**, respectively) and their average length were identified. ROHom and ROHet were grouped according to their length in five different classes: 1 to 2 Mb, 2 to 4 Mb, 4 to 8 Mb, 8 to 16 Mb, and > 16 Mb.

The number of regions (nROHom and nROHet) and their average length across densities were compared using ANOVA.

The ROHom- or ROHet-based coefficients (i.e., **F**_**ROHom**_ and **D**_**ROHet**_) were computed as the ratio between the total sum of ROHom or ROHet length per animal divided by the genome length covered by SNP. As reported by Bordonaro et al. (2023), the D_ROHet_ can be used as coefficient of diversity. The F_ROHom_ and D_ROHet_ values computed in the three different datasets (LD, MD, and HD) were compared using ANOVA.

### Runs of homozygosity and runs of heterozygosity detected on the same animals

To avoid a possible sampling bias, for the animals genotyped at HD, the SNP in common with the LD (43,431 markers) and MD (113,042 markers) datasets were also used to compute both ROHom and ROHet. Moreover, the level of linkage disequilibrium was computed in the three densities to evaluate its potential effect on the runs detection. The squared correlation coefficient of allele frequencies at pairs of loci (*r*2) was estimated for all pairwise combinations of SNP between 0 and 1,000 kb apart (McKay et al., 2007) using Haploview (Barrett et al., 2005).

### Repeated regions and islands

As proposed by Cesarani et al. (2018) and Macciotta et al. (2021), the identified genomic regions starting and ending at the same position found in more than one animal were regarded as repeated (**ROHom**_**REP**_ or **ROHet**_**REP**_) and those in the top 0.1% of the distribution of animals sharing the repeated region were of interest. Finally, the number of animals with an SNP in a run was computed as SNP_ROHom_ and SNP_ROHet_ for the homozygous and heterozygous runs, respectively. The markers exceeding the 99^th^ percentile of these distributions (i.e., **SNP**_**ROHom**_ and **SNP**_**ROHet**_ values) were considered as ROHom or ROHet islands. These two sets of values were compared using ANOVA.

### Gene and quantitative trait loci enrichment

Using the NCBI online database (National Center for Biotechnology Information, www.ncbi.nlm.nih.gov), the genes mapped in or close (± 250 kb; Manca et al., 2020) to the most repeated ROHom and ROHet and to the highlighted islands were identified and described according to the available literature. Moreover, for the same regions, the quantitative trait loci (**QTL**) were identified using the GALLO R package (Fonseca et al., 2020a), which was also used to carry out an enrichment analysis using Bonferroni correction for the *P*-value (0.05).

## Results

### Runs of homozygosity

Results of ROHom, in terms of number of regions and their average length, are reported in Table 1. The number of ROHom per animal showed a non-linear trend: the highest value (47.61 ± 19.80) was observed in the MD and the lowest (16.00 ± 6.52) in LD. On the contrary, the average length decreased by about four times (from LD to HD), as the density of genotyping increased. The same was true for the distribution of ROHom in the different length classes (Table 1): in the HD, only two runs longer than 8 Mb were found. Similar values were observed in the three datasets regarding the average ROHom length within each length class (Table 1). The longest ROHom regions were found on *Bos taurus* (BTA) chromosome 4 (36.90 to 106.38 Mb, 1335 SNP), BTA10 (44.31 to 97.58 Mb, 2301 SNP), and BTA7 (73.62 to 86.88 Mb, 3468 SNP) in the LD, MD, and HD datasets, respectively.

**TABLE 1.** Runs of homozygosity (ROHom) and runs of heterozygosity (ROHet) detected with three densities

Runs	Density^1^	Animals	Runs				Mean ± SD length (number of runs)				
			Total	Unique	Per animal	Average size	1-2 Mb	2-4 Mb	4-8 Mb	8-16 Mb	>16 Mb
ROHom	LD	393	6,288	5,467	16.00 ± 6.52^c^	6.47 ± 5.62^a^	1.40 ± 0.28 (306)	3.14 ± 0.51 (2212)	5.52 ± 1.12 (2356)	10.9 ± 2.24 (1031)	23.60 ± 7.81 (383)
	MD	338	16,091	13,646	47.61 ± 19.80^a^	2.72 ± 2.61^b^	1.46 ± 0.27 (8925)	2.74 ± 0.54 (4648)	5.43 ± 1.10 (1855)	10.80 ± 2.12 (559)	21.10 ± 6.07 (104)
	HD	151	6,404	5,966	42.41 ± 22.70^b^	1.59 ± 0.72^c^	1.33 ± 0.26 (5277)	2.59 ± 0.49 (1028)	4.90 ± 0.89 (97)	11.70 ± 2.21 (2)	—
ROHet	LD	382	1,419	836	3.71 ± 1.89^a^	1.29 ± 0.88^b^	1.26 ± 0.22 (1370)	2.27 ± 0.22 (49)	—		
	MD	217	332	147	1.53 ± 0.72^b^	1.45 ± 0.48^a^	1.26 ± 0.25 (273)	2.35 ± 0.09 (59)	—		
	HD										

^1^LD = low density (50k SNP); MD = medium density (140k SNP); HD = high density (800k SNP).

Different superscript letters within column within ROHom or ROHet indicate significant differences for *P* < 0.001.

**FIGURE 1.**
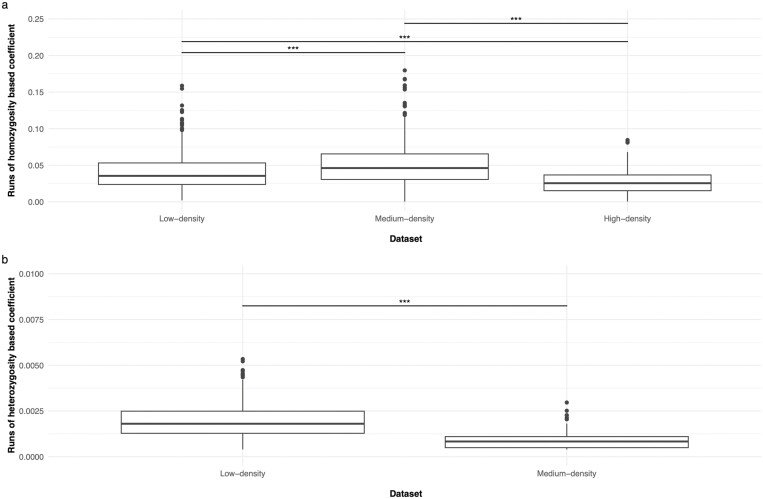
Runs of homozygosity (ROHom) (a) and runs of heterozygosity (ROHet) (b) based coefficients computed in the datasets. Asterisks indicate significant differences for *P* < 0.001.

The F_ROHom_ (i.e., the inbreeding level) computed in LD and MD were quite similar, even if significantly different (*P* < 0.001): 0.04 ± 0.03 (max 0.16) and 0.05 ± 0.03 (max 0.18), respectively (Fig. 1). Lower values were computed for HD (0.03 ± 0.02, max 0.08) because of the lower average ROHom length highlighted in this dataset (Table 1).

### Runs of heterozygosity

In contrast to ROHom detection, the largest nROHet was found in the LD dataset (3.71 ± 1.89), whereas the lowest average nROHet was found in MD (1.53 ± 0.72). The average ROHet length increased (from 1.29 to 1.45 Mb) moving from LD to MD. In the HD dataset, no relevant ROHet were found (Table 1). The ROHet were found only in the first two classes of length (1 to 2 and 2 to 4 Mb), with only 3% (LD) and 18% (MD) of the identified ROHet found in the 2 to 4 Mb length class. As reported in Table 1, the BeadChip density had an impact on the average length of the ROHet, which significantly (*P* < 0.001) increased moving from LD (1.29 ± 0.88 Mb) to MD (1.45 ± 0.48 Mb).

The D_ROHet_ values were very low (all below 1%): the maximum D_ROHet_ observed in LD was 0.005, whereas the maximum value (0.003) was estimated in MD. As shown in Figure 1, the values computed for the LD dataset were significantly larger (*P* < 0.001) than those computed in the MD dataset.

### Runs of homozygosity and runs of heterozygosity detected on the same animals

Results of ROHom and ROHet identified in the 152 animals genotyped with the three densities were reported in Table 2. These results agree, in terms of both the average number of regions per animal and their length, with those observed in the other two groups of animals (Table 1). Moreover, to test a possible effect of the differences in the linkage disequilibrium among the three datasets (LD, MD, and HD), the values of *r*^2^ were compared among different densities (Supplementary Figure S1): no differences were observed in the linkage disequilibrium decay.

**TABLE 2.** Runs of homozygosity (ROHom) and runs of heterozygosity (ROHet) detected with three densities on the same 152 animals

Runs	Density^1^	Total	Unique	Animals	Runs/animal	Average size	Number of runs					Mean ± SD length				
							1-2 Mb	2-4 Mb	4-8 Mb	8-16 Mb	>16 Mb	1-2 Mb	2-4 Mb	4-8 Mb	8-16 Mb	>16 Mb
ROHom	LD	1,778	1,721	148	12.01 ± 5.76^c^	6.03 ± 4.21^a^	36	607	797	276	62	1.47 ± 0.29	3.24 ± 0.47	5.48 ± 1.09	10.80 ± 2.12	21.50 ± 5.89
	MD	7,455	6,735	151	49.37 ± 17.06^a^	2.47 ± 1.95^b^	4,264	2,255	764	153	19	1.46 ± 0.27	2.73 ± 0.55	5.34 ± 1.03	10.40 ± 1.90	19.30 ± 3.35
	HD	6,404	5,966	151	42.41 ± 22.70^b^	1.59 ± 0.72^c^	5,277	1,028	97	2	—	1.33 ± 0.26	2.59 ± 0.49	4.90 ± 0.89	11.70 ± 2.21	—
ROHet	LD	419	339	139	3.01 ± 1.61^a^	1.31 ± 0.30	401	18	—			1.27 ± 0.21	2.34 ± 0.23	—		
	MD	74	50	59	1.25 ± 0.54^b^	1.36 ± 0.30	71	3	—			1.32 ± 0.23	2.29 ± 0.26	—		
	HD	—														

^1^LD = low density (50k SNP); MD = medium density (140k SNP); HD = high density (800k SNP).

Different superscript letters within column within ROHom or ROHet indicate significant differences for *P* < 0.001.

### Repeated regions

In this study, a total of 21 regions exceeding the 0.1% of the ROHom_REP_ distribution and two regions exceeding the 0.1% ROHet_REP_ distribution were found (Table 3). Among the top 21 ROHom_REP_, four were detected in the LD, 13 in the MD, and four in the HD dataset, respectively. The most shared ROHom was found in 27 different animals (~8% of the animals genotyped with the MD BeadChip), and it was located at 38.43 to 39.46 Mb on BTA6 (Table 3), in which no genes were mapped. However, this genomic region overlapped with 414 QTL (Supplementary Table S1). According to the enrichment analysis, a total of 27 different terms were highlighted, of which 13 were significant (Supplementary Table S1). The significant terms were associated with exterior (22 QTL), meat and carcass (114), production (209), and reproduction (4) traits.

**TABLE 3.** Most repeated (i.e., exceeding the 0.1% of the distribution) runs of homozygosity (ROHom) and heterozygosity (ROHet) found in the different datasets

Runs	Density^1^	BTA	Start (bp)	End (bp)	Animals
ROHom	LD	2	189,886	3,046,092	9
		4	49,651,768	50,796,591	13
		5	92,844,631	93,949,810	9
		14	22,983,665	26,473,490	14
	MD	1	3,023,897	4,834,622	10
		3	113,433,557	114,766,179	12
		4	49,760,465	50,900,429	10
		6	37,896,892	39,216,868	10
		6	38,428,952	39,461,621	27
		6	71,044,403	72,475,809	11
		7	41,565,963	43,126,285	13
		11	60,974,044	62,732,451	10
		12	21,352,699	22,885,975	12
		17	55,454,910	56,587,255	11
		18	39,201,407	40,630,538	10
		21	44,800,371	46,145,471	13
		23	15,894	1,580,636	16
	HD	5	12,426,099	13,789,485	6
		6	33,736,732	35,205,727	9
		6	76,922,031	78,084,721	6
		19	90,671	1,333,831	6
ROHet	LD	21	2,151,256	3,245,487	41
	MD	21	173,023	2,504,481	27

^1^LD = low density (50k SNP); MD = medium density (140k SNP); HD = high density (800k SNP).

The most repeated ROHet was found in 41 animals (~10% of the animals in the LD dataset) and it was located at 2.15 to 3.25 Mb on BTA21 (Table 4), where four genes (*SNRPN*, *SNURF*, *UBE3A*, and *ATP10A*) were mapped (± 250 kb downstream and upstream from the repeated ROHet). The same region overlapped with 762 QTL (Supplementary Table S1), which were grouped in 14 terms by the enrichment analysis. Among them, only one, calving ease, was significant (738 QTL).

**TABLE 4.** SNP exceeding the 99th percentile of the number of times a particular SNP was inside a run of homozygosity (SNP_ROHom_) or heterozygosity (SNP_ROHet_) distribution in each density

Runs	BTA	SNP	Position (bp)	QTL^1^	Genes
ROHom	6	31	38,203,273-40,629,318	1,750	*SLIT2*, *PACRGL*, *KCNIP4*
ROHet^2^	16	8	40,880,683-41,345,308	27	*TNFSF18*, *TNFSF4*, *AADACL4*, *DHRS3*, *VPS13D*, *TNFRSF1B*, *TNFRSF8*
	16	1	41,664,706	11	*VPS13D*, *TNFRSF1B*, *TNFRSF8*, *MIIP*, *MFN2*, *PLOD1*, *KIAA2013*, *NPPB*, *NPPA*, *CLCN6*, *MTHFR*
	16	7	42,411,339-43,174,027	24	*DISP3*, *UBIAD1*, *MTOR*, *ANGPTL7*, *EXOSC10*, *SRM*, *MASP2*, *TARDBP*, *CASZ1*, *PEX14*, *DFFA*, *CORT*, *CENPS*, *PGD*, *KIF1B*, *UBE4B*
	16	3	43,501,100-43,697,344	8	*KIF1B*, *UBE4B*, *RBP7*, *NMNAT1*, *LZIC*, *CTNNBIP1*, *CLSTN1*, *PIK3CD*, *TMEM201*, *SLC25A33*
	21	17	2,151,256-3,245,487	762	*SNRPN*, *SNURF*, *UBE3A*, *ATP10A*
	21	1	3,809,287	1	*GABRB3*

^1^Genes and associations with QTL were searched within ± 250 kb downstream and upstream of the reported positions.

^2^Details and references listed in Supplementary Tables S1 and S2.

### ROHom and ROHet islands

**FIGURE 2.**
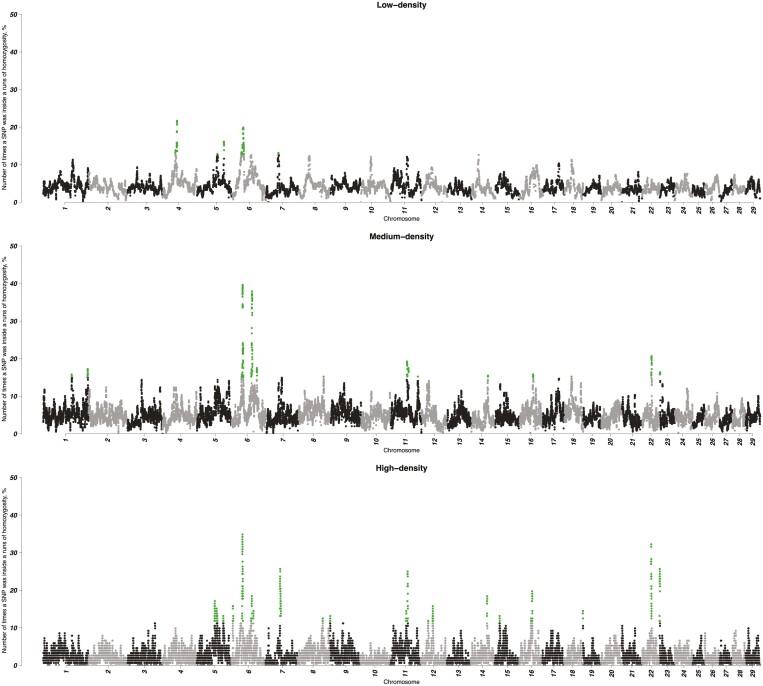
Manhattan plot of the number of times a particular SNP was inside a run of homozygosity (SNP_ROHom_) detected in the three datasets. Green dots represent SNP with a SNP_ROHom_ value exceeding the 99th percentile of its distribution.

**FIGURE 3.**
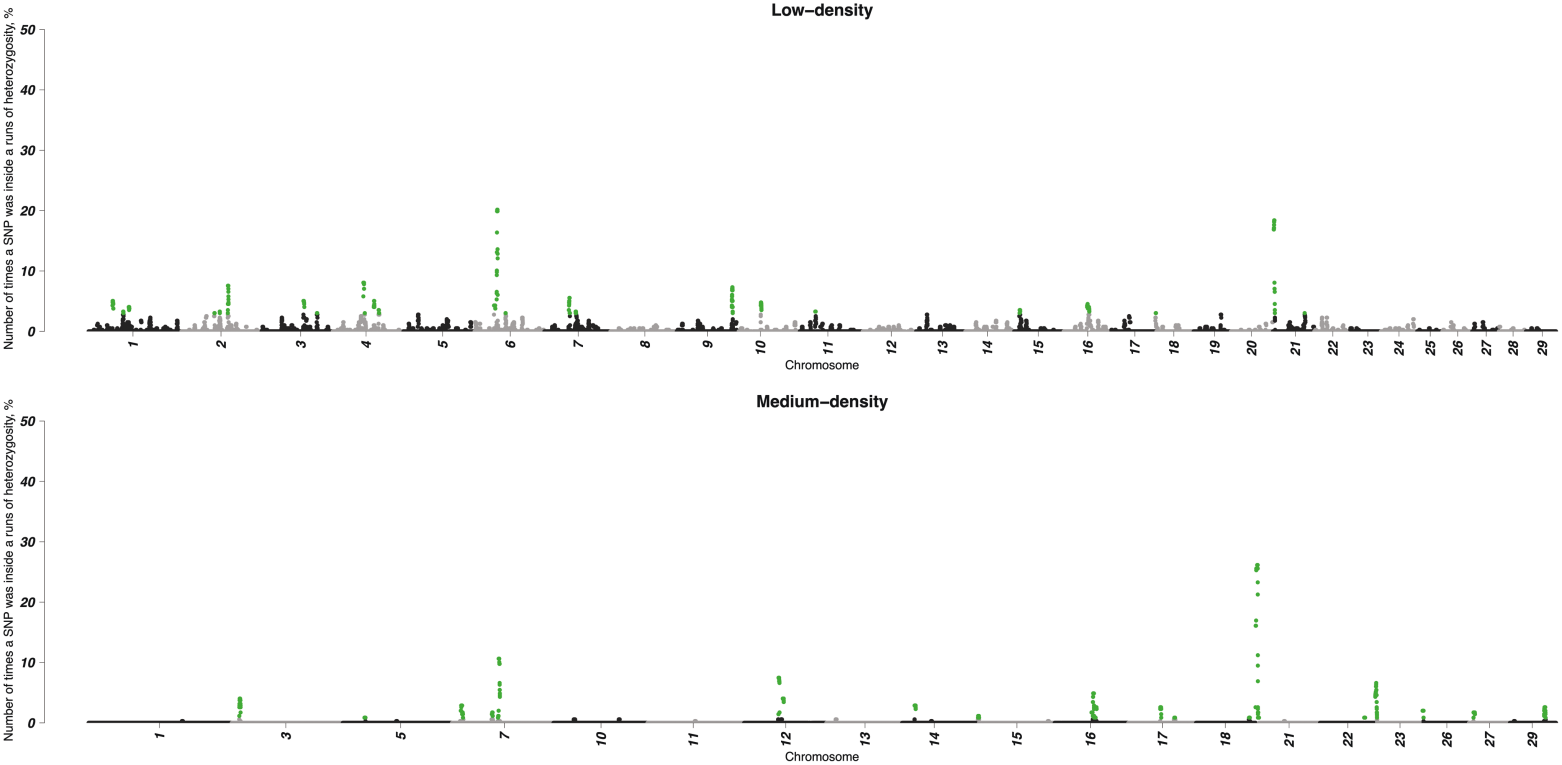
Manhattan plot of the number of times a particular SNP was inside a run of heterozygosity (SNP_ROHet_) detected in the three datasets. Green dots represent SNP with a SNP_ROHet_ value exceeding the 99th percentile of its distribution.

Figures 2 and 3 are Manhattan plots of SNP_ROHom_ and SNP_ROHet_, respectively. Across datasets, the average probability of having a particular SNP inside a ROHom (3.90 ± 2.56%) was significantly higher (*P* < 0.001) compared to ROHet (0.09 ± 0.58%). Thus, an SNP had a larger likelihood to be within an ROHom rather than ROHet. The SNP_ROHom_ values computed in the three datasets were moderately and positively correlated each other: 0.51 (LD vs MD), 0.42 (LD vs HD), and 0.53 (MD vs HD). The correlation between SNP_ROHet_ computed in the LD and MD datasets was lower (0.33).

A total of 419, 1,102, and 5,626 SNP exceeded the 1% of the SNP_ROHom_ distribution in the LD, MD, and HD datasets, respectively: 31 SNP were in common among the three different datasets. These markers mapped on BTA6 between 38.20 and 40.63 Mb and could be considered as one large ROHom island (Table 4). It is interesting to note that this island included the most repeated ROHom (38.43 to 39.46 Mb, Table 3). The genomic region identified by the 31 SNP exceeding the 99th percentile of the SNP_ROHom_ mapped three genes (*SLIT2*, *PACRGL*, and *KCNIP4*) and overlapped with 1,750 QTL. The latter were divided into 61 terms, of which 15 were significant. The significantly enriched terms were related to exterior (22 QTL), meat and carcass (461), production (1,113), and reproduction (4).

A total of 409 (in LD dataset) and 642 SNP (in MD dataset) exceeded the SNP_ROHet_ distribution. There were 37 SNP_ROHet_ in common among the two datasets, of which 19 were mapped on BTA16 and 18 on BTA21 (Table 4). On BTA16, four different ROHet islands were identified; in these regions, a total of 39 genes were present (Supplementary Table S2).

On BTA21, two different islands were highlighted. The longest ROHet island (17 SNP) completely overlapped with the most shared ROHet mentioned above (Table 3). The second peak, highlighted by a single SNP, had one gene, the *gamma-aminobutyric acid type A receptor subunit beta3* (*GABRB3*). The ROHet islands found in the two chromosomes (BTA16 and 21) partially or totally overlapped with 819 QTL (Supplementary Table S1) that were divided in 24 enriched terms. Among them, only 2 were significant: calving ease (738 QTL) and interval to first estrus after calving (20), both in the reproduction group.

## Discussion

The availability of high-throughput sequencing or genotyping SNPs data has opened the possibility of characterizing individual segments of the genome in terms of their homozygosity (regions of homozygosity (ROH)) or heterozygosity (heterozygosity-rich regions) (Biscarini et al., 2020). These genomic regions are both a consequence of the selection that shapes the genomic structure of livestock populations (Bordonaro et al., 2023). The occurrence of ROHet avoids the deleterious effects of continuous homozygous genotypes and favors the heterozygote advantage in immune-related genes as well as in productive and reproductive traits (Sanglard et al., 2021; Chen et al., 2022; Chessari et al., 2024). On the contrary, the study of ROH distribution has been a helpful tool to detect regions potentially under selection (e.g., Mastrangelo et al., 2017; Cesarani et al., 2021; Macciotta et al., 2021). Despite such advancements, both ROHom and ROHet detection, are sensitive to various parameters, such as the genotyping density, and so far, only few studies addressed this issue in ROHom (Purfield et al., 2012; Ferenčaković et al., 2013b; Hillestad et al., 2018) or ROHet (Mulim et al., 2022). Therefore, in this study, the effect of genotyping density on ROHom and ROHet features was tested, and the signals highlighted across datasets were further investigated.

### Runs of homozygosity

The nROHom decreased and their average length increased as the genotyping density increased. This is because a higher SNP density improves the discovery resolution, by reducing the detection of false long ROHom. However, for the same reason, the use of the HD panel could lead to an underestimation of number of segments longer than 8 Mb (Ferenčaković et al., 2013b). This was confirmed by the distribution of ROHom in the different length classes (Table 1). In fact, when a denser SNP panel is used, a larger number of opposite markers (i.e., heterozygote for ROHom and homozygote for ROHet, respectively) can break a long region to shorter ones, thus reducing the number of long regions (Hillestad et al., 2018). On the contrary, Purfield et al. (2012) reported that using an LD BeadChip could lead to an overestimation of regions shorter than 4 Mb. Also, Ferenčaković et al. (2013b) showed that the 50k panel revealed an abundance of small segments and overestimated the numbers of segments 1 to 4 Mb long, suggesting that it is not sensitive enough for the precise determination of small segments. Thus, the LD and HD BeadChips are not able to precisely identify small or long segments, respectively.

To evaluate the detection power of the three investigated datasets, the ROHom statistics were compared with the values reported in the literature for the same breed, to avoid bias due to different breeding management, selection pressure, or evolution history since ROHom patterns can be associated with these phenomena (Purfield et al., 2012; Gaspa et al., 2014; Forutan et al., 2018; Macciotta et al., 2021). Most of the available literature on ROHom detection in this breed involved the use of a 50k SNP, which correspond to the LD dataset in the present study. Most of these studies used an arbitrary fixed number of markers (i.e., 15 SNP) and 1 Mb as minimum values to define a ROHom. For example, Cesarani et al. (2021) and Marras et al. (2015) identified the ROHom in Italian Simmental, and they reported the nROHom as 77.47 ± 15.23 and 94.30 ± 12.20, with an average length of 2.45 ± 3.24 and 2.2 Mb, respectively. Ferenčaković et al. (2011) reported similar values (i.e., nROHom = 96.79 ± 13.37, with an average length of 2.4 Mb) in Austrian Simmental bulls. Finally, Szmatola et al. (2016) found nROHom to be 81.5 ± 11.8 with an average length of 2.49 Mb in Polish Simmental. Zhao et al. (2021) used a HD BeadChip array to investigate ROHom in Chinese Simmental. These authors defined a ROH with at least 100 SNP covering 500 kb and they allowed two homozygous and one missing genotypes. The average nROHom was 99.03, with an average length of 1.18 Mb. All these values, especially regarding nROHom, are greater than those reported here, probably because of the stricter parameters adopted in the present study for ROHom identification (e.g., 50 as minimum number of SNP, and no heterozygotes or missing markers). The MD dataset showed the greatest nROHom (with a standard deviation lower than that reported for HD) and an average size (2.72 ± 2.61 Mb) similar to the values reported in the literature.

The F_ROHom_ values computed in the present study were compared with the estimates reported in literature to investigate which density led to more consistent results. In literature, different F_ROHom_ values are reported for the Simmental breed: 0.08 ± 0.04 (Szmatola et al., 2016), 0.09 ± 0.02 (Ferenčaković et al., 2011), 0.07 ± 0.03 (Cesarani et al., 2021), and 0.08 (Marras et al., 2015). However, values comparable to those estimated in this study were reported in Italian Simmental by Mastrangelo et al. (2018b), with a mean F_ROHom_ of 0.03 ± 0.02. In the present study, the values closer to the values found in the literature were computed using the MD dataset. The lower values here computed could be ascribed to the old age of genotyped animals, especially in the HD. In animal populations under genetic improvement, old animals usually show inbreeding coefficients lower than young animals (Makanjuola et al., 2020; Guinan et al., 2023). Moreover, the number and length of ROHom tend to increase over time: Forutan et al. (2018) analyzed ROHom in North American Holstein cattle and found that the rate of increase of ROHom longer than 1 Mb in the last 5 yr was almost double that of the previous 5-yr period.

Since the LD and HD datasets could lead to bias in the number of detected ROHom and the ROHom statistics (in terms of both average length and inbreeding coefficient) obtained in the MD are closer to the literature, the latter density could represent the best option to detect ROHom in the Simmental breed, also because MD BeadChip is cheaper than HD.

### Runs of heterozygosity

The ROHet identified in the present study were fewer and shorter compared to ROHom. In particular, only ROHet shorter than 4 Mb were found. A similar result was reported in a study on horses, where only ROHet shorter than 2 Mb were identified (Santos et al., 2021). Recently, Chessari et al. (2024) in a study on goats showed an average ROHet length < 1Mb. Moreover, Biscarini et al. (2020) found just two ROHet longer than 2 Mb in Maremmana semi-feral cattle. Ruan et al. (2022) analyzed the distribution of ROHet in two Duroc pig populations and they found only about 3%-5% of regions in the length class > 4 Mb. As already observed for ROHom, the BeadChip density had an impact on both nROHet and their average length, which increased from LD to MD. This result is in agreement with Mulim et al. (2022), who also found shorter ROHet using lower density. Since no ROHet were detected in the HD dataset and the use of LD could overestimate long runs, the MD could represent the optimum array for the ROHet detection, as already pointed out for ROHom. ROHet statistics were not compared with values from the literature, because no studies about ROHet on Simmental cattle were found.

The low D_ROHet_ values computed in the present study are due to the low number of heterozygous regions and their short length. Very low ROHet-based coefficients agree with the few reports available in literature. For example, Bordonaro et al. (2023) analyzed diversity indices estimated from ROHet in pigs and they found average values ranging from 0.0001 to 0.0047. A recent study (Chessari et al., 2024) on ROHet in Italian goat populations reported low values of a similar magnitude (0.0024 ± 0.0003).

### Runs of homozygosity and runs of heterozygosity detected on the same animals

Since the results on ROHom and ROHet were obtained on different datasets of animals, results reported in the present study for different SNP densities may be affected by a sampling bias. To check this hypothesis, analyses were repeated using only the 152 animals genotyped with 800k SNP: LD and MD densities were then mimicked by retaining only the markers included in the lower densities. Results on this subset of animals, in terms of the number of regions and length, were similar to those obtained using the three different datasets. As expected, no differences were observed in the linkage disequilibrium among densities since all animals belonged to the same breed.

### Repeated regions

A genomic region shared among different animals of the same breed could be associated with a selection pressure on portions of the genome that control economically important traits as well as other important animal characteristics such as disease resistance or general immune competence. In particular, if the shared genomic region is characterized by a high level of homozygosity, it could be due to directional selection, both artificial or natural (Kim et al., 2013; Gorssen et al., 2021), whereas a high level of heterozygosity could be associated to balancing selection (Fijarczyk and Babik, 2015).

In the present study, the most shared ROHom was located on BTA6, where no genes were mapped. For the same genomic region, several associations with QTL were found in the enrichment analysis (Supplementary Table S1). The discovery of QTL associated with production and meat and carcass traits was expected due to the breeding goals of this breed.

In the most repeated ROHet, on BTA21, four genes (*SNRPN*, *SNURF*, *UBE3A*, and *ATP10A*) were mapped. The *ubiquitin protein ligase E3A* (*UBE3A*) gene was previously found to be associated with stillbirth and calving ease in cattle (Mészáros et al., 2016). The *ATPase phospholipid transporting 10A, putative* (*ATP10A*) has been associated with calving ease (Frischknecht et al., 2017) and milking speed (Marete et al., 2018). *UBE3A* and *SNRPN* (*small nuclear ribonucleoprotein polypeptide N*) have been associated with cattle temperament by Costilla et al. (2020) and, together with the *SNURF* (*SNRPN upstream open reading frame*) gene, with the age at first calving (Alves et al., 2022). As Suzuki et al. (2009) pointed out, the *SNRPN* and *SNURF* constituted a bicistronic gene (*SNRPN-SNURF*), which has been extensively studied in mice and humans and has been associated with neurodevelopmental disorders. Finally, all four genes were found related to the occurrence of early pregnancy in Nellore cattle (Irano et al., 2016). Also the QTL overlapping with this genomic region were mainly associated with reproduction traits and, in particular, with calving ease. Heterozygosity and, thus, ROHet could be mainly associated with balancing selection rather than directional selection. Indeed, genes and QTL found to be associated with this metric were mostly related to functional phenotypes.

### ROHom and ROHet islands

As shown in Figures 2 and 3, an SNP is more likely to be in an ROHom than an ROHet. This was expected because of the larger number of homozygous genotypes along the genome, which was reflected in the results reported in Table 1. The ROHom island identified by the SNPROHom exceeding the 99th percentile of the distribution in all the three datasets was located on BTA6 and contained three genes. The *slit guidance ligand 2* (*SLIT2*) gene has been previously reported to be involved in several weight traits: in particular, internal organ (especially spleen) weight in Simmental cattle (An et al., 2018), bone weight in beef cattle (Niu et al., 2021), birth, yearling, and weaning weights in US Red Angus cattle (Smith et al., 2022), and birth weight in US Gelbvieh cattle (Smith et al., 2019). Moreover, the same gene was also associated with the infection of tropical theileriosis parasite (Larcombe et al., 2022) and with female fertility in Nordic Red cattle (Höglund et al., 2015). The *parkin coregulated like* (*PACRGL*) has been associated with height and stature of cattle (Doyle et al., 2020). The *potassium voltage-gated channel interacting protein 4* (*KCNIP4*) was reported to be related to milk fat percentage (Pedrosa et al., 2021), fertility (Tarekegn et al., 2021), birth weight and yearling weight (Smith et al., 2022), and to backfat thickness and carcass weight (Srikanth et al., 2020). Moreover, all the three genes were reported to be associated with clinical or subclinical ketosis by Soares et al. (2021). The majority of QTL flagged by this genomic region (Supplementary Table S1) was significantly enriched in two main categories: production—average daily gain, body weight and body weight gain, dry matter intake, metabolic body weight—and meat and carcass—biceps brachii weight, bone weight, carcass weight, lean meat yield, liver weight, longissimus muscle area, and subcutaneous fat thickness. As expected, the ROHom islands harbored genes and QTLs with similar functions than those found in the most repeated ROHom regions. The relationship with meat traits was expected because they have a weight of 24% in the breeding program of Italian Simmental (Cesarani et al., 2020). At the same time, the non-significance of QTLs associated with milk production traits (*N* = 104, Supplementary Table S1) was quite surprising since the latter has a larger weight (44%) in the breeding program of this breed.

Three genomic regions were identified by the SNP_ROHet_, located on chromosomes 16 and 21 (Table 4). Among the genes mapped in BTA16, 11 genes (*SLC25A33*, *PIK3CD*, *CTNNBIP1*, *NMNAT1*, *MTHFR*, *MIIP*, *TNFRSF8*, *TNFRSF1B*, *DHRS3*, *TNFSF4*, and *TNFSF18*) were previously found to be related to reproduction traits, such as early pregnancy, stillbirth, oocyte developmental potential, age at first calving, fertility, and embryo survival (details and references listed in Supplementary Table S2). Ten genes (*TNFSF18*, *TNFRSF1B*, *MFN2*, *PLOD1*, *MASP2*, *DFFA*, *UBE4B*, *CLSTN1*, *CTNNBIP1*, and *PIK3CD*) were reported to be involved with health-related traits: neutrophil response, mastitis, axonopathy, dermatosparaxis, response to *Mycobacterium avium* subsp. *Paratuberculosis*, and retained placenta. Moreover, 12 genes were related to fitness traits such as longevity (*UBIAD1*, *MTOR*, *ANGPTL7*, *EXOSC10*, *SRM*, *MASP2*, *TARDBP*, *CASZ1*) or climate adaptation (*NPPB*, *NPPA*, *MTOR*, *PEX14*, and *CORT*). The only gene mapping in the ROHet island located on BTA21, i.e., *gamma-aminobutyric acid type A receptor subunit beta3* (*GABRB3*), was a candidate gene for temperament traits in a study by Costilla et al. (2020). As already observed for repeated ROHet, genes (Supplementary Table S2) and QTL (Supplementary Table S1) retrieved using runs of heterozygosity were mainly related with functional and reproduction traits.

## Conclusions

In the present study, genotypes from the Italian Simmental cattle breed were used to investigate the impact of BeadChip density in detecting ROHom and ROHet. The results confirmed that the detection of ROHom is more reliable when the array density increases, whereas an opposite trend was observed for ROHet. Moreover, ROHet were not found in HD. Thus, the best option to detect both types of runs could be the use of MD chip. Genes and QTL mapped in the highlighted ROHet were mainly associated with reproduction, health, and fitness traits, whereas the genes and the QTL associated with the ROHom were predominantly involved in meat production traits. The results of the present study strengthened the usefulness of these parameters in investigating these genomic regions and their biological meaning. Further studies are needed on the comparison between these two parameters and deeper analysis of ROHet.

## Supplementary Material

skae147_suppl_Supplementary_Figure

skae147_suppl_Supplementary_Table_S1

skae147_suppl_Supplementary_Table_S2

## Abbreviations

BTA: Bos taurus autosome
*D*_ROHet_: ROHet-based diversity coefficient
*F*_ROHom_: ROHom-based inbreeding coefficient
HD: high-density dataset
LD: low-density dataset
MAF: minor allele frequency
MD: medium density dataset
NCBI: National Center for Biotechnology Information
nROHet: number of ROHet per animal
nROHom: number of ROHom per animal
QTL: quantitative trait loci
ROHet: runs of heterozygosity
ROHet_REP_: repeated ROHet
ROHom: runs of homozygosity
ROHom_REP_: repeated ROHom
SNP: single-nucleotide polymorphisms
SNP_ROHet_: number of times a particular SNP is inside an ROHet
SNP_ROHom_: number of times a particular SNP is inside an ROHom
