# Supplementary figures and images for: Effect of genotyping density on the detection of runs of homozygosity and heterozygosity in cattle
Source: J Anim Sci. 2024 May 27;102:skae147. doi: 10.1093/jas/skae147 (PMC11197001; doi:10.1093/jas/skae147)

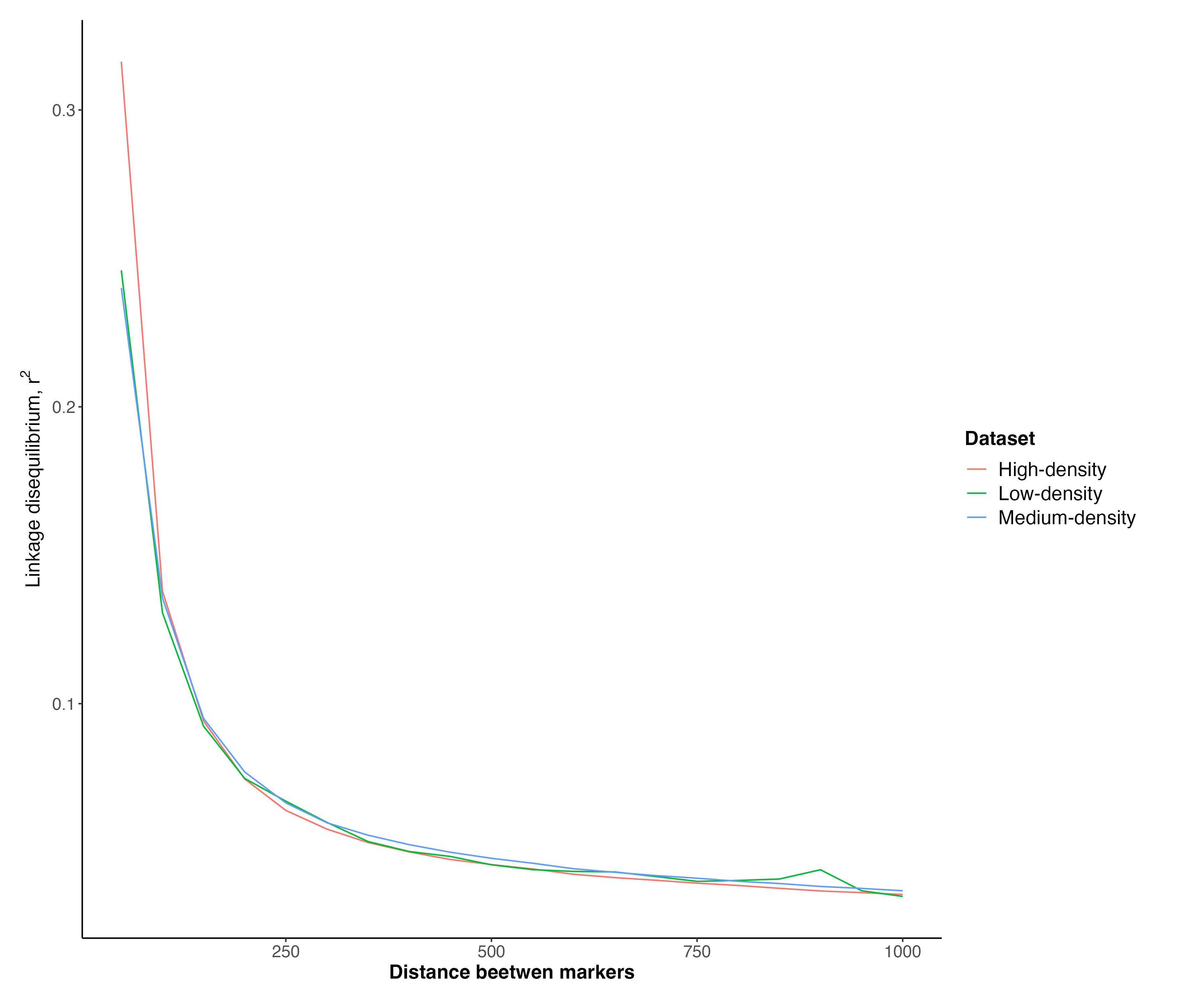

Supplement: skae147_suppl_Supplementary_Figure [file skae147_suppl_supplementary_figure.jpeg]
