# Supplementary material for: Effect of genotyping density on the detection of runs of homozygosity and heterozygosity in cattle
Source: J Anim Sci. 2024 May 27;102:skae147. doi: 10.1093/jas/skae147 (PMC11197001; doi:10.1093/jas/skae147)
Supplement: skae147_suppl_Supplementary_Table_S1 [file skae147_suppl_supplementary_table_s1.docx]

**Table S1**. Results of the quantitative trait loci (QTL) enrichment analysis carried out in the most repeated runs of homozygosity (ROHom) and runs of heterozygosity (ROHet) and in the genomic regions highlighted by the markers exceeding the 99^th^ percentile of the distributions of SNP inside runs of homozygosity (SNP_ROHom_) or inside runs of heterozygosity (SNP_ROHet_). The raw P-values were corrected using Bonferroni. The terms in red are significantly different from zero.

|  | QTL | | | | **P-value** | |
| --- | --- | --- | --- | --- | --- | --- |
| **Parameter** | **Type** | **N** | **Term** | **N** | **Raw** | **Corrected** |
| Most repeated ROHom | Exterior | 26 | **Conformation score** | 16 | 4.44E-27 | 0.00 |
|  |  |  | Dairy form | 1 | 7.28E-01 | 1.00 |
|  |  |  | Digital cushion thickness | 1 | 1.25E-01 | 1.00 |
|  |  |  | **Ear size** | 6 | 3.18E-09 | 0.00 |
|  |  |  | Rump angle | 1 | 3.64E-01 | 1.00 |
|  |  |  | Temperament | 1 | 1.49E-01 | 1.00 |
|  | Health | 3 | Bovine tuberculosis susceptibility | 2 | 7.89E-01 | 1.00 |
|  |  |  | Somatic cell score | 1 | 9.42E-01 | 1.00 |
|  | Meat and Carcass | 117 | **Biceps brachii weight** | 5 | 2.49E-11 | 0.00 |
|  |  |  | **Bone weight** | 71 | 4.01E-108 | 0.00 |
|  |  |  | **Carcass weight** | 21 | 7.77E-08 | 0.00 |
|  |  |  | Connective tissue amount | 1 | 1.00E+00 | 1.00 |
|  |  |  | Lean meat yield | 1 | 7.92E-01 | 1.00 |
|  |  |  | **Liver weight** | 3 | 1.59E-07 | 0.00 |
|  |  |  | **Longissimus muscle area** | 14 | 3.18E-05 | 0.00 |
|  |  |  | Tenderness score | 1 | 1.00E+00 | 1.00 |
|  | Milk | 54 | Curd firming rate | 1 | 7.54E-02 | 1.00 |
|  |  |  | Milk fat percentage | 20 | 9.58E-01 | 1.00 |
|  |  |  | Milk potassium content | 3 | 1.76E-01 | 1.00 |
|  |  |  | Milk protein percentage | 30 | 7.46E-02 | 1.00 |
|  | Production | 209 | **Average daily gain** | 48 | 5.15E-23 | 0.00 |
|  |  |  | **Body weight** | 50 | 4.96E-19 | 0.00 |
|  |  |  | **Body weight gain** | 18 | 1.82E-08 | 0.00 |
|  |  |  | **Dry matter intake** | 28 | 4.63E-12 | 0.00 |
|  |  |  | **Metabolic body weight** | 65 | 2.76E-32 | 0.00 |
|  | Reproduction | 5 | **Calving index** | 4 | 2.76E-06 | 0.00 |
|  |  |  | Male fertility | 1 | 4.93E-02 | 1.00 |
| Most repeated ROHet | Exterior | 2 | Heel horn erosion | 1 | 1.42E-01 | 1.00 |
|  |  |  | Stature | 1 | 9.87E-01 | 1.00 |
|  | Meat and Carcass | 4 | Marbling score | 1 | 1.00E+00 | 1.00 |
|  |  |  | Muscle creatinine content | 1 | 1.97E-01 | 1.00 |
|  |  |  | Muscle potassium content | 1 | 2.08E-01 | 1.00 |
|  |  |  | Shear force | 1 | 1.00E+00 | 1.00 |
|  | Milk | 9 | Milk kappa-casein percentage | 3 | 1.00E+00 | 1.00 |
|  |  |  | Milking speed | 6 | 3.34E-01 | 1.00 |
|  | Production | 5 | Body length | 1 | 9.74E-02 | 1.00 |
|  |  |  | Body weight | 4 | 1.00E+00 | 1.00 |
|  | Reproduction | 742 | Age at puberty | 1 | 1.00E+00 | 1.00 |
|  |  |  | **Calving ease** | 738 | 0.00E+00 | 0.00 |
|  |  |  | Gestation length | 1 | 9.47E-01 | 1.00 |
|  |  |  | Interval to first estrus after calving | 2 | 9.57E-01 | 1.00 |
| SNP_ROHom_ | Exterior | 33 | **Conformation score** | 16 | 3.81E-17 | 0.00 |
|  |  |  | Dairy form | 1 | 9.96E-01 | 1.00 |
|  |  |  | Digital cushion thickness | 1 | 4.34E-01 | 1.00 |
|  |  |  | **Ear size** | 6 | 1.38E-05 | 0.00 |
|  |  |  | Feet and leg conformation | 1 | 9.99E-01 | 1.00 |
|  |  |  | Foot angle | 1 | 9.99E-01 | 1.00 |
|  |  |  | Rump angle | 1 | 8.54E-01 | 1.00 |
|  |  |  | Stature | 1 | 1.00E+00 | 1.00 |
|  |  |  | Strength | 1 | 9.99E-01 | 1.00 |
|  |  |  | Temperament | 1 | 4.97E-01 | 1.00 |
|  |  |  | Udder attachment | 1 | 9.99E-01 | 1.00 |
|  |  |  | Udder depth | 1 | 9.99E-01 | 1.00 |
|  |  |  | Udder height | 1 | 9.96E-01 | 1.00 |
|  | Health | 7 | Bovine respiratory disease susceptibility | 2 | 9.99E-01 | 1.00 |
|  |  |  | Bovine tuberculosis susceptibility | 3 | 1.00E+00 | 1.00 |
|  |  |  | Somatic cell score | 2 | 1.00E+00 | 1.00 |
|  | Meat and Carcass | 475 | **Biceps brachii weight** | 7 | 1.81E-12 | 0.00 |
|  |  |  | **Bone weight** | 87 | 1.02E-84 | 0.00 |
|  |  |  | **Carcass weight** | 168 | 1.45E-93 | 0.00 |
|  |  |  | Connective tissue amount | 1 | 1.00E+00 | 1.00 |
|  |  |  | Fat thickness at the 12th rib | 1 | 8.39E-01 | 1.00 |
|  |  |  | Kidney, pelvic, and heart fat percentage | 1 | 3.90E-01 | 1.00 |
|  |  |  | **Lean meat yield** | 17 | 4.74E-04 | 0.03 |
|  |  |  | **Liver weight** | 3 | 1.19E-05 | 0.00 |
|  |  |  | **Longissimus muscle area** | 136 | 3.39E-81 | 0.00 |
|  |  |  | Marbling score | 1 | 1.00E+00 | 1.00 |
|  |  |  | Muscle magnesium content | 1 | 3.63E-01 | 1.00 |
|  |  |  | Muscle phosphorus content | 1 | 3.13E-01 | 1.00 |
|  |  |  | Muscle potassium content | 1 | 4.15E-01 | 1.00 |
|  |  |  | Muscle sodium content | 1 | 4.52E-01 | 1.00 |
|  |  |  | Shear force | 1 | 1.00E+00 | 1.00 |
|  |  |  | Spleen weight | 3 | 1.60E-03 | 0.10 |
|  |  |  | **Subcutaneous fat thickness** | 43 | 5.39E-25 | 0.00 |
|  |  |  | Tenderness score | 2 | 1.00E+00 | 1.00 |
|  | Milk | 104 | Curd firming rate | 1 | 2.83E-01 | 1.00 |
|  |  |  | Milk C18 index | 1 | 9.29E-01 | 1.00 |
|  |  |  | Milk fat percentage | 34 | 1.00E+00 | 1.00 |
|  |  |  | Milk fat yield | 2 | 1.00E+00 | 1.00 |
|  |  |  | Milk potassium content | 4 | 8.58E-01 | 1.00 |
|  |  |  | Milk protein percentage | 58 | 1.00E+00 | 1.00 |
|  |  |  | Milk protein yield | 1 | 1.00E+00 | 1.00 |
|  |  |  | Milk stearic acid content | 1 | 9.04E-01 | 1.00 |
|  |  |  | Milk yield | 2 | 1.00E+00 | 1.00 |
|  | Production | 1,120 | **Average daily gain** | 302 | 5.64E-189 | 0.00 |
|  |  |  | Body depth | 1 | 9.97E-01 | 1.00 |
|  |  |  | **Body weight** | 129 | 3.18E-25 | 0.00 |
|  |  |  | **Body weight gain** | 47 | 4.71E-12 | 0.00 |
|  |  |  | **Dry matter intake** | 209 | 1.03E-128 | 0.00 |
|  |  |  | Length of productive life | 1 | 1.00E+00 | 1.00 |
|  |  |  | **Metabolic body weight** | 426 | 1.4532E-288 | 0.00 |
|  |  |  | Net merit | 1 | 1.00E+00 | 1.00 |
|  |  |  | PTA type | 1 | 9.99E-01 | 1.00 |
|  |  |  | Residual feed intake | 2 | 9.38E-01 | 1.00 |
|  |  |  | Rump width | 1 | 9.96E-01 | 1.00 |
|  | Reproduction | 11 | Calving ease | 2 | 1.00E+00 | 1.00 |
|  |  |  | **Calving index** | 4 | 7.14E-04 | 0.04 |
|  |  |  | First service conception | 1 | 9.97E-01 | 1.00 |
|  |  |  | Inseminations per conception | 1 | 1.00E+00 | 1.00 |
|  |  |  | Interval from first to last insemination | 1 | 9.92E-01 | 1.00 |
|  |  |  | Male fertility | 1 | 1.93E-01 | 1.00 |
|  |  |  | Non-return rate | 1 | 1.00E+00 | 1.00 |
| SNP_ROHet_ | Exterior | 3 | Heel horn erosion | 2 | 1.19E-02 | 0.29 |
|  |  |  | Stature | 1 | 9.91E-01 | 1.00 |
|  | Health | 10 | Bovine respiratory disease susceptibility | 1 | 9.85E-01 | 1.00 |
|  |  |  | Somatic cell score | 9 | 1.15E-01 | 1.00 |
|  | Meat and Carcass | 9 | Lean meat yield | 1 | 9.56E-01 | 1.00 |
|  |  |  | Marbling score | 2 | 9.99E-01 | 1.00 |
|  |  |  | Muscle creatinine content | 1 | 2.10E-01 | 1.00 |
|  |  |  | Muscle potassium content | 1 | 2.21E-01 | 1.00 |
|  |  |  | Shear force | 2 | 1.00E+00 | 1.00 |
|  |  |  | Subcutaneous fat thickness | 2 | 7.22E-01 | 1.00 |
|  | Milk | 28 | Milk fat percentage | 7 | 1.00E+00 | 1.00 |
|  |  |  | Milk fat yield | 2 | 1.00E+00 | 1.00 |
|  |  |  | Milk kappa-casein percentage | 3 | 1.00E+00 | 1.00 |
|  |  |  | Milk protein percentage | 8 | 1.00E+00 | 1.00 |
|  |  |  | Milk protein yield | 1 | 1.00E+00 | 1.00 |
|  |  |  | Milking speed | 7 | 2.47E-01 | 1.00 |
|  | Production | 9 | Body length | 1 | 1.04E-01 | 1.00 |
|  |  |  | Body weight | 6 | 1.00E+00 | 1.00 |
|  |  |  | Body weight gain | 1 | 9.99E-01 | 1.00 |
|  |  |  | Metabolic body weight | 1 | 1.00E+00 | 1.00 |
|  | Reproduction | 760 | Age at puberty | 1 | 1.00E+00 | 1.00 |
|  |  |  | **Calving ease** | 738 | 0.00E+00 | 0.00 |
|  |  |  | Gestation length | 1 | 9.57E-01 | 1.00 |
|  |  |  | **Interval to first estrus after calving** | 20 | 5.90E-07 | 0.00 |
