# Supplementary material for: Effect of genotyping density on the detection of runs of homozygosity and heterozygosity in cattle
Source: J Anim Sci. 2024 May 27;102:skae147. doi: 10.1093/jas/skae147 (PMC11197001; doi:10.1093/jas/skae147)
Supplement: skae147_suppl_Supplementary_Table_S2 [file skae147_suppl_supplementary_table_s2.docx]

**Table S2**. List of the genes mapped in the ROHet islands.

| **BTA** | **Position (Mb)** | **Gene acronym** | **Associated traits** | **Reference** |
| --- | --- | --- | --- | --- |
| 16 | 40.65-40.66 | TNFSF18 | Neutrophil response  Embryo survival | Wathes et al., 2021  Zolini et al., 2020 |
| 16 | 40.82-40.84 | *TNFSF4* | Pregnancy | Mamo et al., 2012 |
| 16 | 41.06-41.08 | *AADACL4* | – | |
| 16 | 41.11-41.16 | *DHRS3* | Residual feed intake  Pregnancy | Chen et al., 2011  Hughes et al., 2022 |
| 16 | 41.21-41.48 | *VPS13D* | – | |
| 16 | 41.50-41.53 | *TNFRSF1B* | Fertility  Mastitis | Fonseca et al., 2020b  Wang et al., 2016 |
| 16 | 41.56-41.64 | *TNFRSF8* | Pregnancy  Milk fat percentage | Pedrosa et al., 2021  Mamo et al., 2012 |
| 16 | 41.66-41.67 | *MIIP* | Fertility | Fonseca et al., 2020b |
| 16 | 41.68-41.71 | *MFN2* | Degenerative Axonopathy in Tyrolean Grey Cattle | Drögemüller et al., 2011 |
| 16 | 41.72-41.75 | *PLOD1* | Dermatosparaxis | Gorjidooz et al., 2021 |
| 16 | 41.74-41.76 | *KIAA2013* | Meat quality | Leal-Gutiérrez et al., 2020 |
| 16 | 41.823-41.824 | *NPPB* | Cold adaptation  Intramuscolar fat | Cardona et al., 2014  Strucken et al., 2017 |
| 16 | 41.83-41.84 | *NPPA* | Cold adaptation  Marbling score | Cardona et al., 2014  Lim et al., 2013 |
| 16 | 41.84-41.87 | *CLCN6* | Carcass and growth traits in chicken  Milk production ability in pigs | Zhang et al., 2020  Shi et al., 2021 |
| 16 | 41.87-41.89 | *MTHFR* | Fertility  Milk fat synthesis | Fonseca et al., 2020b  Fedota et al., 2018  Zhou et al., 2019 |
| 16 | 42.15-42.20 | *DISP3* | Carcass and growth traits in chicken  Milk production ability in pigs | Zhang et al., 2020  Shi et al., 2021 |
| 16 | 42.37-42.39 | *UBIAD1* | Longevity | Steri et al., 2019 |
| 16 | 42.39-42.52 | *MTOR* | Longevity  Heat tolerance | Steri et al., 2019  Ning et al., 2019 |
| 16 | 42.447-42.453 | *ANGPTL7* | Longevity  Marbling fat | Steri et al., 2019  Hudson et al., 2020 |
| 16 | 42.52-42.55 | *EXOSC10* | Longevity | Steri et al., 2019 |
| 16 | 42.56-42.57 | *SRM* | Longevity  Milk protein synthesis | Steri et al., 2019  Menzies et al., 2009 |
| 16 | 42.58-42.60 | *MASP2* | Longevity  Mastitis resistance | Steri et al., 2019  Zhang et al., 2019 |
| 16 | 42.59-42.61 | *TARDBP* | Longevity  Body weight gain | Steri et al., 2019  Lindholm-Perry et al., 2020 |
| 16 | 42.80-42.96 | *CASZ1* | Longevity | Steri et al., 2019 |
| 16 | 42.96-43.11 | *PEX14* | Climate adaptation  Heat stress response | Liu et al., 2021  Singh et al., 2020 |
| 16 | 43.11-43.12 | *DFFA* | Response to *Mycobacterium avium* subsp. *paratuberculosis* | Kabara et al., 2010 |
| 16 | 43.127-43.129 | *CORT* | Cold climate adaptation | Shen et al., 2020 |
| 16 | 43.13-43.15 | *CENPS* | – | |
| 16 | 43.16-43.17 | *PGD* | – | |
| 16 | 43.19-43.34 | *KIF1B* | – | |
| 16 | 43.48-43.50 | *UBE4B* | Mastitis resistance | Grossi et al., 2014 |
| 16 | 43.53-43.56 | *RBP7* | Back fat thickness in pigs | Zhang et al., 2021 |
| 16 | 43.57-43.60 | *NMNAT1* | Age at first calving | Mota et al., 2017 |
| 16 | 43.60-43.62 | *LZIC* | Residual feed intake in broilers | Li et al., 2021 |
| 16 | 43.63-43.68 | *CTNNBIP1* | Oocyte developmental potential  Retained placenta | Walker and Biase, 2020  Guarini et al., 2019 |
| 16 | 43.71-43.79 | *CLSTN1* | Retained placenta | Guarini et al., 2019 |
| 16 | 43.79-43.85 | *PIK3CD* | Stillbirth  Mastitis  Feed efficiency | Martin et al., 2022  Khan et al., 2022  Taye et al., 2018 |
| 16 | 43.89-43.91 | *TMEM201* | – | |
| 16 | 43.92-43.95 | *SLC25A33* | Pregnancy | Cerri et al., 2012 |
| 21 | 1.94-1.96 | *SNRPN* | Cattle temperament  Early pregnancy  Age at first calving | Costilla et al., 2020  Alves et al., 2022  Irano et al., 2016 |
| 21 | 1.94-1.95 | *SNURF* | Early pregnancy  Age at first calving | Alves et al., 2022  Irano et al., 2016 |
| 21 | 2.30-2.40 | *UBE3A* | Stillbirth and calving ease  Cattle temperament  Early pregnancy  Age at first calving | Mészáros et al., 2016  Costilla et al., 2020  Alves et al., 2022  Irano et al., 2016 |
| 21 | 2.76-2.94 | *ATP10A* | Calving ease  Milking speed  Early pregnancy | Frischknecht et al., 2017  Marete et al., 2018  Irano et al., 2016 |
| 21 | 3.87-4.15 | *GABRB3* | Temperament | Costilla et al., 2020 |
